# Tissue Engineering Through 3D Bioprinting to Recreate and Study Bone Disease

**DOI:** 10.3390/biomedicines9050551

**Published:** 2021-05-14

**Authors:** Adriene Pavek, Christopher Nartker, Maamoon Saleh, Matthew Kirkham, Sana Khajeh Pour, Ali Aghazadeh-Habashi, Jared J. Barrott

**Affiliations:** 1 Department of Biomedical and Pharmaceutical Sciences, Idaho State University, Pocatello, ID 83209, USA; paveadri@isu.edu (A.P.); nartchri@isu.edu (C.N.); kirkmatt@isu.edu (M.K.); khajsana@isu.edu (S.K.P.); 2 Whitman College, Walla Walla, WA 99362, USA; salehmm@whitman.edu

**Keywords:** 3D bioprinting, bone, osteoporosis, heterotopic ossification, osteosarcoma, Paget’s disease, osteogenesis imperfecta, rickets disease

## Abstract

The applications of 3D bioprinting are becoming more commonplace. Since the advent of tissue engineering, bone has received much attention for the ability to engineer normal bone for tissue engraftment or replacement. While there are still debates on what materials comprise the most durable and natural replacement of normal tissue, little attention is given to recreating diseased states within the bone. With a better understanding of the cellular pathophysiology associated with the more common bone diseases, these diseases can be scaled down to a more throughput way to test therapies that can reverse the cellular pathophysiology. In this review, we will discuss the potential of 3D bioprinting of bone tissue in the following disease states: osteoporosis, Paget’s disease, heterotopic ossification, osteosarcoma, osteogenesis imperfecta, and rickets disease. The development of these 3D bioprinted models will allow for the advancement of novel therapy testing resulting in possible relief to these chronic diseases.

## 1. Introduction

Skeletal bone, which supports and protects whole body organs, is a biologically active and vascularized connective tissue. Bone remolding and turnover is a dynamic process through which, sequential bone-resorption and bone-formation, the new bone tissue replaces the old tissue [1]. Bone has self-healing abilities to some extent. However, it is not able to repair if the imbalance between bone-resorption and bone-formation causes major defects because of some diseases such as osteoporosis [2], Paget’s disease [3], and cancer [4], or bone fractures due to significant trauma. This issue highlights bone transplantation as the most common therapeutic option. Every year globally, millions of bone grafts are transplanted in surgical procedures to treat bone defects. Based on Polaris market research, the bone graft industry will be worth about $4.15 billion by 2026. Although different types of autologous, allogeneic, and xenogeneic bone grafts have their advantages, this approach is not without limitations and disadvantages [5]. Risk of surgical complications, graft tissue rejection due to immunogenic response, and infection are some complications to mention. Recently, to address these obstacles, alternative bone graft materials obtained via bone tissue engineering have gained significant attention [6]. Through a multidisciplinary field of biomaterial engineering and medicine, scientists aim to regenerate damaged or lost bone tissues through the association of cells, scaffolds, and biological agents. Bone tissue 3D scaffolds provide a proper environment for cell attachment, proliferation, differentiation, and new tissue regeneration. Biocompatible, non-toxic synthetic and natural polymers with proper durability, mechanical strength, and high surface area offer good candidates for bone tissue engineering scaffolds for different therapeutic goals other than bone and tissue repair, such as novel drug delivery [7].

3D printing of biological material is a rapidly growing field of interest. A new and complex area of 3D bioprinting is replicating human bone. Producing a 3D model of diseased human bone with living cells is of particular interest for testing novel drug therapies. Such implications provide the opportunity to test drug therapies in an environment that closely represents the in vivo situation and reduces the requirements for extensive animal and human testing. Testing novel drugs on 3D tissue versus 2D tissue are essential as the latter fail to represent the in vivo environment [8]. There are several methods for 3D printing of the bone, all using different materials. Few articles have been published on how to bioprint 3D diseased bone. This review article contemplates six common disorders of the bone: osteoporosis, Paget’s disease, heterotopic ossification, osteosarcoma, osteogenesis imperfecta, and rickets disease; and how they could be replicated utilizing 3D bioprinting for future novel drug testing. Before discussing these disease states, a brief summary of normal bone printing is provided.

## 2. Process of Printing Bone Tissue

The process for bioprinting bone tissue has many promising implications for biomedical research. Whether one is engineering bone for tissue transplant or is recapitulating the unique biology of bone, the processes are similar [9]. However, despite the transformative potential this technology possesses, there are still many considerations that one must take prior to bioprinting normal bone tissue.

The bioprinting process begins by designing a scaffold. Designing a scaffold entails constructing a structure of the desired bone tissue that mimics the physical components of the ECM (extracellular matrix) [10]. The properties of the surrounding physical environment include, but are not limited to, the stabilizing structured support or scaffold, biochemical/cellular interactions for the cells that will soon comprise the printed tissue, and proper access to nutrients throughout the printed bone tissue. These properties can be manipulated by changing the scaffold’s shape, porosity, and chemical composition [11].

Once an appropriate scaffold has been designed, the selection or creation of the bioink follows. Bioinks are generally considered to be the liquid mixture of cells and other biocompatible materials that will then be deposited into the designed scaffold. The purpose of the bioink is to provide cell support and nutrients that promote proliferation and differentiation to develop a more accurate model of the desired bone tissue [12]. The noncellular component of bioinks can fall into three broad categories:

- bioceramic powders;
- hydrogels; and
- other polymers synthesized from raw materials.

The composition of the bioink is heavily dependent on the printing technique and the desired scaffold that the scientists are seeking [13]. Other components of bioink include structural proteins such as collagen and other growth factors vital to the healthy development of the cells in the printed tissue.

Once the composition of the bioink is determined, then the bioink is embedded in the scaffold. There are many ways to embed the bioink into a scaffold, such as laser-based methods, ink-jet based methods, and other CAD (computer aided design) programmable methods [13]. The best approach will depend on the composition of the bioink, the tools accessible to the scientist, and the environment in which the printing process will take place. When embedding bioink for bone tissue, it must take place on the outer perimeter of the scaffold while containing a decreasing concentration of calcium as it reaches the inner center of the scaffold. This step is done to create the proper localization of the cancellous and cortical parts of the bone. The cortical bone, accounting for roughly 10% of the bone, is located on the peripheral parts of the bone, and the cancellous—consisting anywhere from 50–90% of the bone—is surrounded by the exterior cortical region of the bone. To promote continued growth at the center of the bone, one must ensure that the scaffold has interconnected pores that can reach the center of the scaffold, and provide vital nutrients and growth factors. This highlights the importance of the parameters that constitute the scaffold. Properties, such as the size of the pores, in the scaffold, are essential constructs to consider when printing any tissues.

The porosity of the scaffold can be constructed using many techniques in lieu of additive manufacturing. These techniques include chemical/gas foaming, solvent casting, foam gel, and freeze-drying [13]. The limitations to all the approaches mentioned above are the lack of control over parameters that contribute to the properties of porosity for the given scaffold. To have greater control over such parameters, additive manufacturing techniques provide a promising alternative for tissue scaffold engineering. Some examples of additive manufacturing are 3D printing, solid freeform fabrication, and rapid prototyping [10]. Nearly all of these additive manufacturing techniques utilize computationally designed models consisting of information for the structure, porous properties, and organizational composition of the scaffold.

The last step in the process of printing bone tissue is to combine the bioink material with the scaffold design. In designing scaffolds, there are inorganic scaffolds that are biodegradable, materials used for bone mineralization, and approaches mimicking natural bone structure. A critical step in engineering bone diseases is to fabricate a bone structure that can mimic the structure of the native bone tissue. To accomplish this aim, several materials can be used. In this review, we will explore several materials that have a high potential to help accomplish this purpose.

## 3. Materials for Printing Bone Tissue

To engineer a bone tissue, the knowledge of cells, biomaterials, and biochemical factors should be combined. As a result, the newly manufactured scaffold should provide a structure for cells to attach and proliferate, which is one definition of biocompatibility. In the case of bone bioprinting the scaffold should be mechanically robust, while a flexible bioscaffold is recommended for cartilage regeneration. Simultaneously, these scaffolds should allow for cell adhesion and bony tissue in-growth. The printing materials that are most advantageous in terms of strength, pliability, and osteoconductivity are often combinations of polymers, plastics, metals, or hydroxyapatite. Each material type is reviewed individually below and summarized in Table 1.

### 3.1. Polymers

Polymers can be selected and engineered for their unique properties that mimic different aspects of bone biology and are often combined to achieve the most relevant recapitulation of bone physiology. Polymers that can be manipulated by environmental stimuli are also highly desirable and reviewed elsewhere [14]. Choosing a polymer that can change properties or degrade in response to temperature, photons, magnetism, chemicals, or humidity allows for dynamic construction of the variegated layers of the bone. Different layers of the structure can be designed for bone bioprinting, including: (i) a first layer of a biodegradable polymer, (ii) a second layer of bone material, and (iii) a third layer of biodegradable polymer deposited on the second layer, each layer repeating until a 3D printer has completed the layered bone graft. Alternatively, a hybrid PCL-collagen scaffold has been manufactured using a two-step process. Poly(ε-caprolactone) or PCL is the most common polymer used for 3D bone tissue engineering. While it exhibits an exceptional transition temperature and melting temperature suitable for extrusion in 3D bioprinting, it is highly hydrophobic, which decreases the surface compatibility for cell adhesion [33]. To enhance PCL applications and cell adhesion, PCL and collagen struts have been stacked after fabrication and demonstrated an appropriate pore size for bone tissue substitution with higher biological activity for osteoblasts in comparison with a plain PCL scaffold [34].

While PCL is easy to use and approved for clinical use, there are alternative polymers that can offer superior biological applications, but still have unknowns about their biocompatibility and safety, making them a risky application for clinical use. Poly(lactic acid) or PLA has an acceptable melting point for material extrusion in 3D printing and, due to its natural origins, it is biodegradable [16,35]. Another polymer-based option is poly(vinyl alcohol) or PVA, a water-soluble filament used for extensive support applications. PVA and PLA have similar melting temperatures [15,36], an important feature when printing two filaments simultaneously.

### 3.2. Metals, Glasses, Plastic, and Ceramics

Potentially any material that can be melted, passed through an extruder, and solidified soon after printing at room temperature or on cooling surfaces can be used in bioprinting. In clinical settings, thermoplastics and waxes have been used, but metals and ceramics are acceptable choices as well [23,37].

Substitution of the bone in craniomaxillofacial surgery has been achieved using different techniques in different bones of the body such as mandible, maxilla, orbital, and nasal bones [38]. For example, patients affected by orbital wall fracture received a 3D-printed pre-bent titanium mesh [39], or in one case study, a mandible was printed using super-thin layers (16 µm) of hard plastic and a gel-like support material to achieve similar reconstruction of bones with intricate geometries [40]. Using hard plastics and metals in 3D printing is advantageous over traditional reconstruction methods like stereolithography due to the increased resolution, faster time to construct, and lower cost [40].

Recently, bio-glasses were tested in 3D bone manufacturing and showed a high bioactivity, osteogenesis, and angiogenesis of bone cells in orthopedics implants [41]. Another 3D-bioprinted nanocomposite material consisting of copper-loaded-ZIF-8 nanoparticles (PLGA/Cu(I)@ZIF-8) was prepared as an antibacterial and osteoconductive scaffold and suggested as a construct for bone infection repair [21]. PLGA (poly-lactide-co-glycolic acid) by itself is a suitable thermoplastic that is FDA-approved and offers a scaffold with high superior strength, but comes with the downside of requiring high temperatures for extrusion. Titanium scaffolds incorporating niobium have also been fabricated, which significantly enhanced the biocompatibility by increasing the surface hydrophilicity [22].

### 3.3. Apatite Derivatives

**Figure 1:**
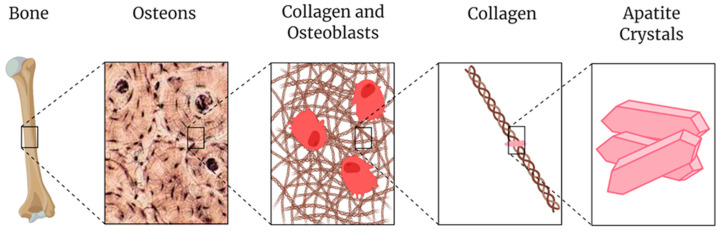
The multi-scale structure of natural bone (from left to right: bone, osteons [45], osteoblasts in collagen, collagen, and apatite crystals). Adapted figure from Kolodziejska et al. [44]

Currently, the main biomaterials for fabricating porous scaffolds are the minerals hydroxyapatite and calcium phosphate due to their high biocompatibility and biodegradability. However, because of low mechanical strength, most such-printed scaffolds are only used in non-load bearing regions [42]. Due to the complexity of microporous structures in conventional bone graft materials, they lack suitability for spontaneous bone generation. As a result, cells cannot fully penetrate and grow. An interesting approach to overcome this problem in printing the bone scaffold is to use apatite and its derivatives, which are also present in natural bone structure (Figure 1). Novel titanium-apatite hybrids were used for spinal applications and the scaffold showed desirable properties for osteoblasts to grow, adhere, and keep their morphology [43]. Bone formation and maturation were also investigated by different formulations of apatite bioprints including carbonate apatite, hydroxyapatite, and β-tricalcium phosphate in the form of honeycomb granules. Among all of the aforementioned compositions, carbonate apatite showed 100% bone maturation degree [32]. The major components of a natural bone composite are type I collagen and nanocrystalline-substituted hydroxyapatite, which are currently used in orthopedic surgeries, but with the limiting factors of fragility and poor mechanical strength as mentioned previously. However, the combination of collagen/apatite is a good choice for having high biocompatibility and allowing osteoblasts to grow and proliferate and should be a high consideration for any combination blends. Using a chitosan-calcium phosphate ink, Caballero et al. successfully achieved a bone substitute by printing a suspension of dispersed calcium phosphate particles in a chitosan acidic aqueous solution with intertwined particles of apatite. This approach made a suitable scaffold for bioprinting bone [27]. In addition to being highly biocompatible, the porous structure allows the bioprint to act as both a bone-filler and a possible option for a drug-delivery system by releasing the drug in a controlled manner directly to the site of the action targeting bone diseases. The collagen/apatite composites have been tested in both in vivo and in vitro studies and demonstrated effectiveness as drug carriers and reservoirs [44].

Further considering bone acting as a bed releasing the drug, Liu et al. tested a porous polycaprolactone/hydroxyapatite (PCL/HA) scaffold, modified with vascular endothelial growth factor (VEGF). This scaffold was analyzed for its effects on cell adhesion, proliferation, and vascularized bone regeneration [46]. Other materials that have been fabricated and introduced as bone are: silver nanoparticles (AgNPs) trapped into the polydopamine (pDA) layer, apatite [47], poly(glycolic acid)/hydroxyapatite [48], and nano-hydroxyapatite (nHAp) polyoxymethylene composites on 316L stainless steel [49]. All these composites have shown good cellular compatibility.

### 3.4. Cell-Material Interactions in Bone Tissue Bioprinting

The interplay between cells and biomaterials is a fundamental topic in 3D bioprinting for bone tissue engineering. The biomaterials’ topographical and chemical surface stimuli can impact cellular behavior at the interface. The process of osteogenic regeneration is comprised of different stages that need to occur. From that point of view, cellular activities most influenced by biomaterial properties are adhesion, spreading, migration, proliferation, and differentiation [50]. For the occurrence of these processes, cellular communication is essential. Therefore, a clear understanding of biology involving cells, ECM, growth factors, ions, and their interactions with biomaterials are pivotal in bone tissue engineering.

3D scaffolds as a central part of bone bioprints are biocompatible constructs similar to ECM that provides mechanical support for in vivo cell attachment and bone tissue formation through biomedical cross-talk and mechanical interaction. These interactions profoundly influence cell behavior, leading in some cases to a cellular network that results in the so-called cellular bridge [51]. As an initial step in the cellular differentiation process, the formation of such a cellular bridge provides strong evidence for cell-scaffold biocompatibility. It necessitates particular physicochemical properties that scaffolds should possess. The scaffold’s pore size, pore volume, and mechanical strength are critical parameters that define its performance. The bone ingrowth initially happens at the marginal section of scaffolds, and then the interconnected pores facilitate continuous ingrowth of bone tissue. This mechanically strong network of pores allows nutrients and molecules to transport to inner parts of a scaffold to promote cell ingrowth, vascularization, and waste material removal. In later stages, the biodegradation process through dissolution or a cell-mediated reaction modulates the replacement of scaffold by a new bone structure [52].

Despite limited available information, the complex interaction between modified biomaterials and cell behavior has not yet been fully explored. Hence, there is a strong need for further investigations to determine the physicochemical properties and influential parameters that reflect the cell physiological behavior upon cell interaction with the biomaterial surface.

## 4. Bone Diseases

There are several bone diseases described by abnormal osteocyte activity resulting in altered bone structure and density. In this section, we briefly discuss the pathophysiology, current drug treatments, and most recent available 3D bioprinting approaches for the management of these diseases.

### 4.1. Osteoporosis

Osteoporosis is a bone disease characterized by reduced bone density, which could diminish the bone microarchitectural strength and put the patient at higher risk of bone fractures [2]. Fractures could occur when a high enough mechanical load, such as trauma, is applied to osteoporotic bone. These fractures are chronic in nature and could cause disability, and their frequency increases with age. Osteoporosis can be either primary or secondary in nature. Primary osteoporosis can occur in both genders and is associated with increased age, however, secondary osteoporosis is a consequence of other diseases or a side effect of some medications [2]. In the United States, more than 10 million individuals over 50 years of age have osteoporosis [2]. Its prevalence is expected to increase as more than 50% of the population over the age of 50 will have, or be at risk of developing, osteoporosis by 2025. Fractures caused by osteoporosis were estimated at 9 million in the year 2000 globally, and mainly affects the elderly population aged 60 and over [40,41]. With the aging world population, osteoporosis will have substantial individual, societal, and economic consequences in the US and internationally.

Osteoporosis is characterized by a low bone density which is 2.5 times less dense than the young-adult mean [53]. Its general pathophysiology involves an increase in osteoclast activity causing an increase in bone resorption and a decrease in osteoblast activity resulting in a decrease of new bone formation [54]. Many of the cytokine pathways involved include CX3CL/CX3CR1 activity, which has shown a positive correlation with osteoporosis development and increase in inflammatory cytokines, such as TNF-α, IL-1B, and IL-6, as well as inflammatory factors found in blood serum TRACP-6b and NTx [55]. Another proposed cytokine pathway for osteoporosis development is bone resorption and deposition primarily regulated by the receptor activator of nuclear factor kappa-B ligand (RANKL) which is produced by osteoblasts and binds to RANK on an osteoclast precursor cell, thereby activating it. Once activated, the osteoclast precursor forms a multinucleated cell which becomes an osteoclast [53]. Additionally, bone turnover markers can be assessed for selection of treatment and short-term monitoring (see Table 2 for list of bone turnover markers) [56].

**Figure 2:**
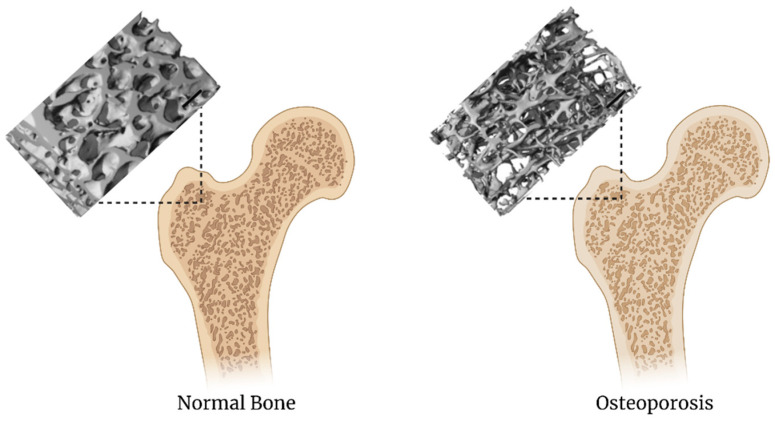
Comparison of normal bone to osteoporotic bone. MicroCT images adapated from Boerckel et al. [59] The scale bar is 1 mm

There are many causes for osteoporosis therefore treatment comprises determining the cause and tailoring treatment on an individual basis [57]. There are several classes of therapeutic agents currently used to treat and prevent osteoporosis. These classes include potent nitrogen-containing bisphosphonates, selective estrogen receptor modulators, hormone replacement therapy, and biologic compounds such as Denosumab (monoclonal antibody), parathyroid hormone, and calcitonins [35]. Lack of potency and increased adverse event and toxicity concerns with these agents necessitate alternative, novel therapeutic and delivery options. Utilizing 3D bioprinting, an osteoporotic bone could potentially be recreated if the 3D bioprinter was fine-tuned enough to print a bone that has an internal structure 2.5× less dense than that of a regular 3D bioprinted bone (Figure 2) [58]. Additionally, active osteoblasts and osteoclasts in their respective ratios associated with osteoporosis can be incorporated into the 3D printing scheme so that therapeutic applications can evaluate the balancing act between osteoblast and osteoclast activity. Thus, 3D bioprinting can achieve a model of osteoporosis in vitro by either creating a scaffold with a reduction in minerals or increased porosity. Biocompatibility can be achieved by including PLGA and its derivatives in the bioink so that both osteoblasts and osteoclasts can be co-cultured on the scaffold. Drugs can then be applied to restore the delicate balance.

3D bioprinting has been shown to help manage osteoporosis disease. 3D printed porous titanium (pTi) alloy scaffolds were combined with pulse electromagnetic fields (PEMF) in vivo to enhance bone regeneration and osseointegration in osteoporosis [60]. This strategy promotes osseointegration of artificial prosthesis or dental implants for patients with osteoporosis. 3D printed Ti6A14V scaffolds have also been combined with freeze dried platelet-rich plasma as a bioactive interface for enhancing osseointegration in osteoporosis [61]. 3D printed polydopamine-coated poly-(lactic-co-glycolic acid)/β-tricalcium phosphate composite scaffolds were able to improve osteogenesis through the increase of polydopamine concentrations [62]. This could be used to help repair defects in the bone from osteoporosis, tumors, or trauma.

### 4.2. Paget’s Disease

**Figure 3:**
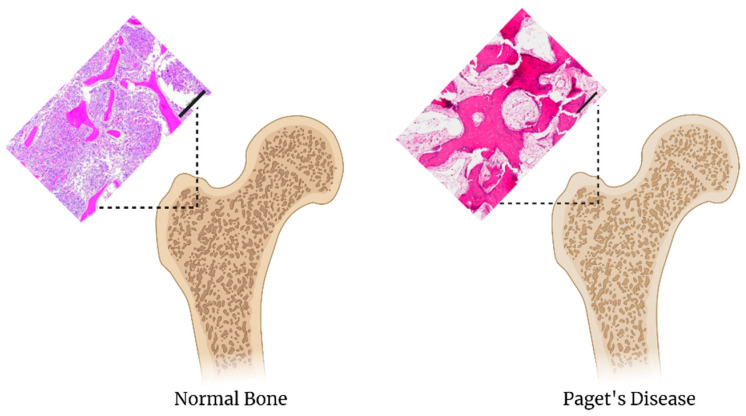
Comparison of normal bone to Paget’s disease The H&E stain of the normal bone scale is 200 um, images adapated from [66]. The H&E stain of Paget’s Disease scale bar is 100 um, images adapated from [67].

Paget’s disease is similar to osteoporosis in that osteoclasts become abnormally activated, leading to an increase in bone resorption [63]. The osteoclasts are increased in number and size, moreover, pagetic osteoclasts are more responsive to osteoclastogenic factors such as TNF-α and RANKL [64]. Osteoblast activity differs in Paget’s from osteoporosis because osteoblasts in Paget’s become activated to compensate for osteoclast activity but end up creating a disorganized mosaic of new bone formation. This disorganized bone molding causes a person to experience pain, bone deformations, arthritis, and an increased risk of bone fracture [64]. Paget’s disease involves a number of genetic factors including the TNFRSF11A and TNFRSF11B genes which encode for RANK and osteoprotegerin (OPG), respectively. Other putative gene mutations linked to Paget’s disease are SQSTM1, VCP, HNRNPA1, HNRNPA2B1, MATR3, TIA1, and ZNF687 and a tangential link to the overexpression of c-Fos, which is partially responsible for the development and progression of osteosarcoma [62,64,65]. Signaling pathways commonly involved in Paget’s disease are NFkB, IKB, RANK, and RANKL [65]. The exact trigger for Paget’s disease is still unknown. Treatment consists of aminobisphosphonates, such as zoledronic acid [63,64]. Similar to recreating the bone microenvironment for osteoporosis, a less dense, yet disorganized scaffold can be created. Again, it is important to use scaffold components that are bioinductive and bioconducive to co-culturing osteoblasts and osteoclasts. Utilizing 3D bioprinting, a Paget’s disease bone could potentially be recreated if the 3D bioprinter was fine-tuned enough to print a bone that is rugged in shape as compared to that of a regular 3D bioprinted bone (Figure 3).

β-tricalcium phosphate (β-TCP)/PLGA scaffold loaded with an osteogenesis promoting drug, HA15 has been used to promote osteogenesis and bone regeneration in defective bone conditions like Paget’s disease, rickets disease, and osteogenesis imperfecta [68]. This scaffold enhanced angiogenesis, reduced apoptosis, and induced autophagy in vivo. Various calcium phosphate (CaP) bioceramics have been used for bone regeneration in disease states [69]. The calcium phosphate component of the bioceramic includes monocalcium phosphate monohydrate (MCPM), dicalcium phosphate anhydrous (DCPA), dicalcium phosphate dehydrate (DCPD), octocalcium phosphate (OCP), hydroxyapatite (HA), Fluorapatite (FA), monocalcium phosphate anhydrous (MCPA), α-tricalcium phosphate (α-TCP), β-tricalcium phosphate (TTCP), or tetracalcium phosphate (TTCP). The commercially available calcium orthophosphate cements include those that have apatite, carbonated apatite, or Brushite [69]. As substitutes of calcium phosphate, calcium carbonates, calcium sulfates, bioactive glasses, and composite materials combining bioactive inorganic materials with biodegradable polymers are some of the most promising biomaterials for application in bone regeneration.

### 4.3. Heterotopic Ossification

**Figure 4:**
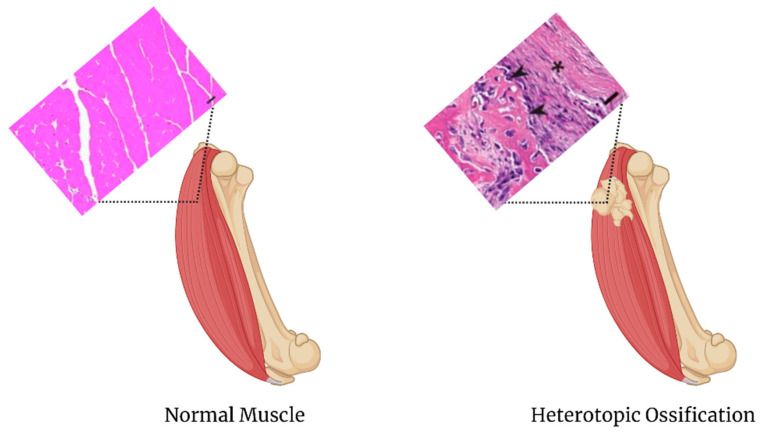
Comparison of normal bone to Heterotopic Ossification. The normal muscle H&E stain scale bar is 40 um, images adapated from [73]. The heterotopic ossification H&E stain scale bar is 25 um, images adapated from [74].

Heterotopic ossification (HO) is the formation of bone outside normal bone growth areas and most commonly occurs in muscle and joint tissue (Figure 4). This abnormal bone formation can commonly be caused by cancer, bone injury, genetic dispositions, surgery, burns, electrocution, fractures, and neurological damage. HO is also a hallmark of the rare fibrodysplasia ossificans progressiva (FOP) genetic disorder [70]. Mesenchymal cell recruitment, proliferation, and differentiation into chondrocytes followed by osteoblast activation and osteoprogenitor maturation are required for ectopic bone formation [71,72].

There are many molecular pathways and factors involved in the microenvironment of the development of heterotopic ossification. Some key cellular markers include: BMP, which is a member of TGF-β signaling pathway; mTOR, which is a target of rapamycin and is believed to play a key role in HO formation; and AMP activated protein kinase [72]. Other possible cellular markers include ebselen, inhibitory ICs (Pd1, PD-L1, and CD152), and stimulatory ICs (CD40L, OX-40L) [72].

Hypoxia inducible factor 1α (HIF-1α) is another key cellular marker of HO [75]. HIF-1α is a subunit of HIF-1 and works with the HIF-1β subunit to regulate the body’s response to hypoxia. Under normal conditions HIF-1α is ubiquitinated and degraded in the cytoplasm. However, under hypoxic conditions HIF-1α is transferred to the nucleus and forms an active complex with HIF-1β. HIF-1 functions as a transcription factor and regulates over 100 genes involved in stem cell proliferation, differentiation, angiogenesis, and bone formation. HIF-1α is expressed in the early stages of heterotopic ossification [75].

New blood vessels develop before the development of HO. Thus, high expression of vascular factors is necessary for the formation of HO. Vascular factors that have been observed in early HO include SOX-9, Runx-2, and VEGF [75]. The process involving these factors is speculated as following: hypoxic environment leads to high levels of HIF-1α, which promotes expression of SOX-9 and causes development of chondrocytes. Then HIF-1α uses the BMP/SMAD pathway to regulate the expression of Runx-2, promoting osteoblast formation. VEGF amplifies the BMP/SMAD pathway, causing heterotopic ossification [75].

Current treatment for heterotopic ossification only involves post development of the disease since prophylaxis need is difficult to foresee [70]. After the heterotopic bone has formed, it needs to be surgically removed [70]. Options for treatment include indomethacin, NSAIDs, low-dose radiation, and BMP receptor inhibitors [71].

Molecular pathophysiology associated with HO initiation is poorly understood, however, understanding the signals and pathways for transformation are essential in reversing or removing HO pharmacologically. Thus, printing mesenchymal cells that can transform to bone upon inflammatory signaling is an important first step in establishing a model sufficient for drug testing. As stated earlier, there are several parallels between bone cancer initiation and HO, especially in their association with inflammation. A recent study of a soft-tissue sarcoma demonstrated that this tissue can ectopically form bone similar to what is seen in HO [76]. The most likely hypothesis is that a mesenchymal stem cell with the potential for bone differentiation is triggered by the presence of immune cells and their release of paracrine cytokine factors.

While there are many factors contributing to mesenchymal stem cells differentiating into HO, there are some basic signaling molecules that are consistently observed in the conversion of mesenchymal cells to HO. Transforming growth factor beta-1 (TGFβ) is associated with HO in chondrogenic progenitor cell differentiation [77].

In an attempt to reduce HO in vivo, Daly et al. included 3D printed microchannels. This strategy including endochondral bone tissue engineering supports the bone healing process with lower level of heterotopic bone formation with the microchanneled templates supporting the lowest levels of heterotopic bone formation. Taken together they showed that using the 3D bioprinted hypertrophic cartilage grafts they could successfully present a method for repairing the fracture without further complications, such as development of HO [78].

### 4.4. Osteosarcoma

**Figure 5:**
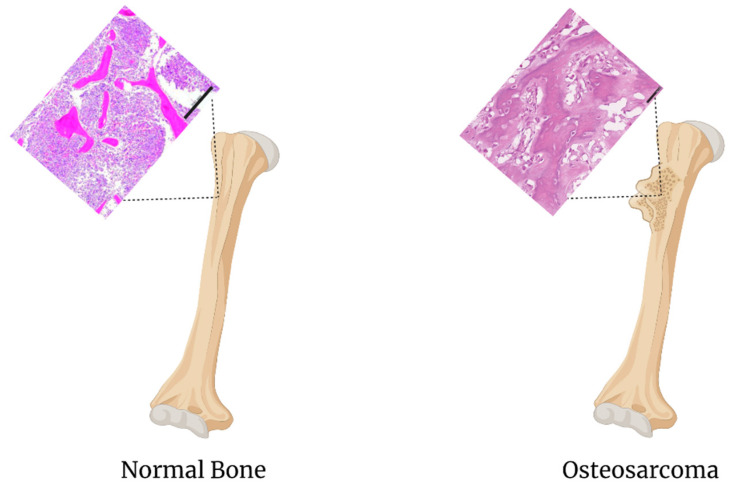
Comparison of normal bone to osteosarcoma. The H&E stain of the normal bone scale bar is 200 um, images adapated from [66]. The H&E stain of the osteosarcoma scale bar is 50um, images adapated from [82].

Osteosarcoma is a malignant bone tumor primarily affecting the long bones in the body (Figure 5) [79]. Each year, there are approximately 1000 new cases of osteosarcoma in the United States. Half of these cases are found in children and teens, and approximately 2% of childhood cancers are osteosarcoma [80]. Worldwide, there is an estimated incidence rate of 5 million cases per year [81].

While there are many pathways for osteosarcoma to develop, it is believed that development comes mainly when genetic or epigenetic changes affect osteoblast differentiation from mesenchymal stem cells [83]. Many of the potential biomarkers thought to be involved in osteosarcoma include increased miR-9 expression, which positively correlates with tumor progression, microRNA 21, NFkB, RelA/RelB, CSF2/GM-CSF, CSF3/G-CSF, BMP2, CCL5, CXCL5, CXCL1, IL6, IL8, CXCR4, IRX1, CXCL14/NF-KB, UPR (GRP78, endoplasmin, calreticulin, and prelamin-A/C), and BMP-2 [79,84,85,86,87,88].

Genes contributing to the pathogenesis of osteosarcoma include downregulation or deletion of RB1, tP53, WWOX, LOX, and KLF6 [81]. Genes contributing to the proliferation of osteosarcoma include dysregulation of APEX1, MDM2, and c-Myc. MMPs, P13Ks, and Cyr61 are involved in metastasis of osteosarcoma [81]. Genes contributing to angiogenesis of osteosarcoma include VEGF, HIF-1a, STAT3, and mTOR [81]. Genes related to apoptosis and proliferation in osteosarcoma are AURKA, YB-1, ROCK1, and MK. MicroRNAs that are downregulated in osteosarcoma are miR-132, miR-451, miR-133a, miR-218, miR-195, and miR-223 [81]. MicroRNAs that are upregulated in osteosarcoma are miR-17, miR-33a, and miR-215 [81]. Genes contributing to osteoclast function in osteosarcoma include RANKL/MCP-1 and RUNX2 [81].

Osteosarcoma is currently treated with surgery and chemotherapy. When using a multidisciplinary approach, the five-year survival rate ranges from 60–70% [89]. However, there are still 30–40% of patients who are poor responders to the standard of care therapy. A burgeoning field in cancer pharmacology is the ex vivo phenotypic screening of a patient’s cancer [90]. This method employs a drug panel to test against the patient’s tumor divided into miniaturized experiments. Patient-derived organoids are increasingly used in this drug screening throughput assay, sometimes referred to as functional precision medicine [91]. For osteosarcoma, resected tumor material can be used to print the patient-derived organoids. This can be performed on a scaffold of normal bone and with the patient’s autologous peripheral blood mononuclear cells (PBMCs). Combining co-culture bioprinting of cancer cells and immune cells will allow for the ex vivo identification of cancer immunotherapies, for which osteosarcoma is a potential beneficiary [92].

Additionally, 3D bioprinting has been utilized to treat osteosarcoma. 2D Black-Phosphorous (BP) nanosheets integrated into 3D printed bioglass (BG) scaffold have been used to treat osteosarcoma [93]. This BP-BG scaffold combines the photothermal properties of BP and the phosphorus and calcium biomineralization of the BP nanosheets to have an improved therapeutic outcome in situ. Alternatively, 2D Ti_3_C_2_ MXene with 3D bioactive glass has used photothermal hyperthermia to simultaneously eradicate osteosarcoma tumors and promote bone regeneration on in vivo xenografts [94]. Post-surgical resection of a bone tumor, Cu-TCPP nanosheets interface-structured β-tricalcium phosphate (TCP) scaffold, when activated with a near-infrared light (NIR), killed remaining osteosarcoma cells and supported the attachments of stem cells [95]. 3D printed polydopamine-coated poly-(lactic-co-glycolic acid)/β-tricalcium phosphate composite scaffolds were able to improve osteogenesis through the increase of polydopamine concentrations. This could be used to help repair defects in the bone from osteoporosis, tumors, or trauma [62].

### 4.5. Osteogenesis Imperfecta

**Figure 6:**
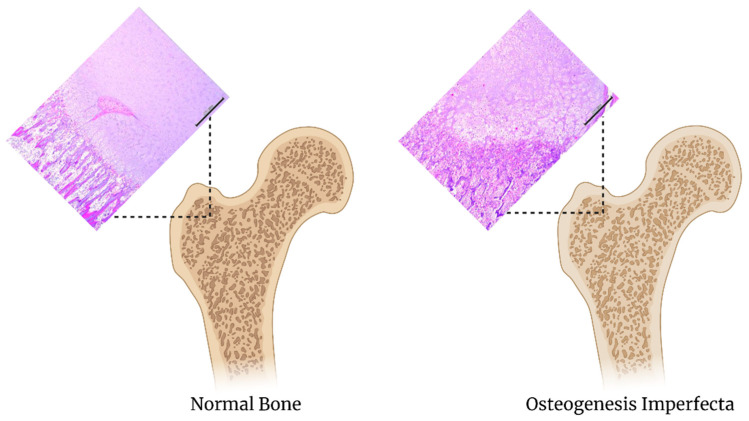
Comparison of normal bone to osteogenesis imperfecta. The scale bar is 200 um, images adapated from [66].

Osteogenesis imperfecta, also known as brittle bone disease, is a genetic disorder that affects the connective tissue found in bones and throughout the body (Figure 6) [96]. Most of the mutations that occur impair collagen synthesis and modifications leaving the patient with bones that easily fracture [97]. There are currently seven types of osteogenesis known [98]. The commonly affected genes in osteogenesis imperfecta include COL1A1, COL1A2, CRTAP, LEPRE1, PPIB, or P3H1 [97,99,100]. The drugs used to treat osteogenesis imperfecta include bisphosphonates such as pamidronate, or zoledronic acid, and denosumab [98,99].

3D organoid models of bone to study osteogenesis have been explored by guiding the differentiation of human bone marrow stromal cells that give rise to both osteoblasts and osteoclasts [100]. Not only was the scaffold and media culture conditions important in stimulating osteogenesis and mineralized ECM production, but a calibrated level of sheer stress was important in the bone formation. Once differentiated, these osteocytes exhibited the normal characteristics of bone as evidenced by the expression of sclerostin and mineralized matrix. The organoid formation of woven bone in this case is very similar to the aberrant bone formation that occurs in osteogenesis imperfecta, right down to the natural co-assembly of collagen fibrils and mineral bone matrix as determined by Raman micro-spectroscopy [100].

Mentioned in the Paget’s disease section, Β-TCP/PLGA scaffold loaded with HA15 promotes osteogenesis and bone regeneration in defective bone conditions like osteogenesis imperfect [68]. CaP bioceramics have also been used for bone regeneration [69].

### 4.6. Rickets Disease

**Figure 7:**
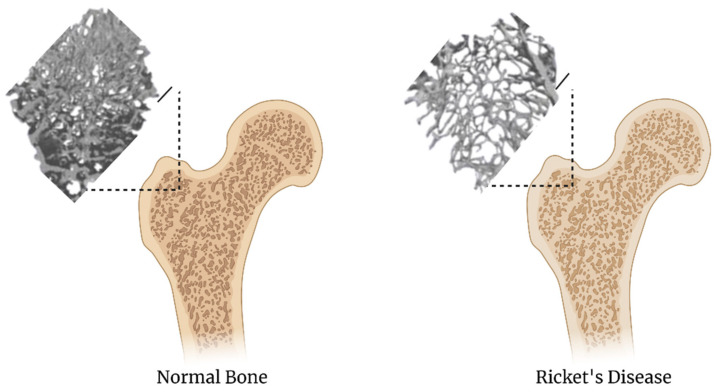
Comparison of normal bone to rickets disease, images adapated from [105]. The scalebar is 1 mm

Rickets disease also known as osteomalacia is impaired mineralization and ossification of the growth plates of growing children due to deficiency of vitamin D (Figure 7) [101]. It usually occurs in three stages. Stage 1 is development of deficiency in vitamin D, causing hypocalcemia. Second-degree hyperparathyroidism also develops which increases renal calcium reabsorption and phosphate excretion. Stage 2 is characterized by the normalization of circulating calcium, while parathyroid hormone and alkaline phosphatase become elevated causing hypophosphatemia, and this hypophosphatemia is thought to cause a failure in chondrocyte apoptosis. Stage 3 is characterized by decreasing calcium absorption leading to hypocalcemia and worsening 2°HPTH [102]. Hereditary hypophosphatemic rickets with hypercalciuria is a rare autosomal recessive disorder [103]. Treatment usually involves increasing calcium and vitamin D supplementation, but with chronic untreated osteomalacia the bones can become weakened and may require other interventions. Additionally, anti-FGF23 antibodies are becoming alternative therapies to reduce the over production of FGF23 signaling [104]. Recreating osteomalacia by 3D bioprinting will allow for the testing of therapies that can stimulate bone density resulting in bone strengthening.

Mentioned in the Paget’s disease and osteogenesis imperfecta section, Β-TCP/PLGA scaffold loaded with HA15 promotes osteogenesis and bone regeneration in defective bone conditions like rickets disease [68]. Various CaP bioceramics can also be used to regenerate bone in rickets disease [69].

## 5. Conclusions

While this is a brief review of potential applications for the future of 3D bioprinting of various bone diseases, it serves to highlight the various directions and applications that are yet to be explored. Particularly in the area of bone printing, there are still many details and specifications to be determined for a single condition, let alone the six diseases discussed above and diseases beyond the scope of this review. As more advances are discovered in 3D bioprinting technologies, this field is projected to expand exponentially.

It is noteworthy to mention that the six bone disease states have significant overlap in their pathophysiologies and undertaking a serious study of any one of these will lead to impacts in others. For example, if a model for osteoporosis is being developed in which drugs are being tested to activate osteoblasts and bone formation, this might result in imperfect bone formation, which could lead to a new disease model for Paget’s disease, or osteogenesis imperfecta. Similarly, heterotopic ossification can be modeled by programmed differentiation of mesenchymal stem cells, which could shed light regarding the early events associated with transformation of bone cells or mesenchymal stem cells that result in osteosarcoma. These disease models are interwoven with subtle nuances that require fine-tuning of the cells, scaffolds, bioinks, and signaling molecules.

Modeling of diseased bone states that can then be manipulated in vitro has the potential to lead to high-throughput screening for treatment analysis to correct bone deficiencies. As proper modeling and printing conditions are determined, the inclusion of actual diseased bone cells, i.e., osteosarcoma cells, will also allow for the exploration of genetic therapies to reverse or target malignant cancer cells. Determination of proper growth factors or genetic manipulations will lead to an improved quality of life for those suffering from these conditions, particularly the elderly population and children who have to live in fear of simple falls and traumas.

The most promising aspect of bioprinting is the potential for a 3D printable bone graft that can be applied in a surgical setting to help with healing in either trauma or resection operations. Bioink material preloaded with suitable growth factors holds the potential to significantly decrease recovery times. Most promising would be a print-in-place application where the bone graft can be applied while printing. Models have already been utilized to evaluate cartilage regrowth in joint and bone wounds using mesenchymal stem cells in a hyaluronic acid-gelatin methacrylate hydro-gel that can then differentiate and replicate as deemed biological prudent [106]. Even if an in situ biological scaffold proves problematic, a transplantable graft would also be a significant improvement for patient quality of life and recovery.

## Figures and Tables

**Table 1:** Bone scaffold material properties.

Material	Biocompatibility	Resolution	Tm	Derivatives and Blends	Ref
PCL	+++		50–60 °C	HA, VEGF, TCP	[14]
PVA	+++	50 µm	220–240 °C		[15]
PLA	+		130–180 °C		[14,16]
PDA			130–180 °C	AgNP	[17]
PEOT/PBT	+++		225 °C		[14]
POM	++	10 µm	232 °C	nHAp, EVA	[18]
PEEK	+/+++		350 °C	HA, AgNPs, pDA	[14,19]
PLGA	+++	~300 µm	240–280 °C	Cu(I)	[20,21]
Titanium	++	40 µm	1670 °C		[22]
PMMA	+	50 µm	160 °C		[23,24]
ABS plastic	-		190 °C		[14,23]
PLLA		50 µm	170–180 °C		[25,26]
CCP	++	30 µm	1670 °C	TCP, BMP-2, nHAP, HA, DCPD	[27,28,29,30,31]
Carbonate Apatite	+++	~2 µm	1000 °C	HA	[32]
HOA	++	115 µm	1100 °C	TCP, AgNPs	[32]

(+) denotes how well cells can adhere to the surface of the scaffold.

**Table 2:** Biomarkers of Bone Turnover.

Bone Biomarker	Sample Location
Osteocalcin	Serum
Bone alkaline phosphatase	Serum
Type I procollagen-C-propeptide	Serum
Intact type I procollagen-N-propeptide	Serum
Total type I procollagen-N-propeptide	Serum
Bone resorption markers:	
Pyridinoline	Urine
Deoxypyridinoline	Urine
Type I collagen cross-linked N-telopeptide	Serum/Urine
Type I collagen cross-linked C-telopeptide	Serum/Urine
Type I collagen C-telopeptide	Serum
Tartrate-resistant acid phosphatase 5b	Serum
Bone matrix-related markers:	
Undercarboxylated osteocalcin	Serum
Pentosidine	Plasma/Urine
Homocysteine	Plasma

## Data Availability

Not applicable.

## Abbreviations

α-TCP	α-tricalcium phosphate
ABS	acrylonitrile butadiene styrene
AMP	adenosine monophosphate
APEX1	apurinic/apyrimidinic endodeoxyribonuclease 1
AURKA	aurora kinase A
β –TCP	β-tricalcium phosphate
BG	bioglass
BP	black phosphorous
BMP	bone morphogenic protein
C-X-C	C-X-C chemokine receptor type 4
CXCL1	C-X-C motif chemokine ligand 1
CX3CL	C-X3-C motif Chemokine ligand 1
CXCL5	C-X-C motif chemokine ligand 5
CX3CR1	C-X3-C motif Chemokine Receptor 1
CXCL14	C-X-C motif chemokine ligand 14
CCL5	C-C motif chemokine 5 precursor
CRTAP	cartilage associated protein
Cyr61	cysteine-rich angiogenic inducer 61
c-Myc	cellular myeloctyomatosis
CD40L	cluster of differentiation 40 ligand
CD152	cluster of differentiation 152
COL1A1	collagen type 1 alpha 1 chain
COL1A2	collagen type 1 alpha 2 chain
CAD	computational aided design
DCPA	dicalcium phosphate anhydrous
DCPD	dicalcium phosphate dehydrate
ECM	extracellular matrix
FA	Fluorapatite
FOP	fibrodysplasia ossificans progressiva
CSF3/G-CSF	granulocyte colony stimulating factor 3
CSF2/GM-CSF	granulocyte macrophage colony stimulating factor 2
HNRNPA1	heterogeneous nuclear ribonucleoprotein A1
HNRNPA2B1	heterogeneous nuclear ribonucleoproteins A2/B1
HIF-1α	hypoxia inducible factor 1α
HA	hydroxyapatite
IL-1B	interleukin 1 beta
IL-6	interleukin 6
IL-8	interleukin 8
IKB	international klein blue
IRX1	Iroquois homeobox 1
KLF6	krupple like factor 6
LEPRE1	leucine proline-enriched proteoglycan 1
LOX	lysyl oxidase
mTOR	mechanistic target of rapamycin
MATR3	matrin 3
MMPs	matrix metalloproteinases
MK	midkine
MCPA	monocalcium phosphate anhydrous
MCPM	monocalcium phosphate monohydrate
MDM2	mouse double minute 2 homolog
NTx	naltrexone
nHAp	nano-hydroxyapatite
NIR	near infrared light
NSAIDs	nonsteroidal anti-inflammatory drugs
NFkB	nuclear factor kappa-light-chain-enhancer
OCP	octocalcium phosphate
PPIB	peptidylprolyl isomerase B
PI3K	phosphoinositide 3-kinase
PAR	photoreactive acrylic resin
PBMCs	peripheral blood mononuclear cells
PCL	polycaprolactone
PDA	phenylenediamine
pDA	polydopamine
PLA	polylactic acid
PLGA	poly-lactide-co-glycolic acid
PVA	polyvinyl alcohol
PCL/HA	porous polycaprolactone/hydroxyapatite
PLGA	polylactic-co-glycolic acid
pTi	porous titanium
PD1	programmed cell death protein 1
PD-L1	programmed death-ligand 1
P3H1	prolyl 3-hydroxylase 1
PEMF	pulse electromagnetic felds
RANKL	receptor activator of nuclear factor kappa-B ligand
RB1	retinoblastoma 1
ROCK1	rho-associated coiled-coil-containing protein kinase 1
Runx-2	runt-related transcription factor
SQSTM1	sequestosome 1
SOX9	SRY-Box Transcription Factor 9
STAT3	signal transducer and activator of transcription 3
AgNPs	silver nanoparticles
TRACP-6b	tartrate-resistant acid phosphatase isoform 6b
TTCP	tetracalcium phosphate
TIA1	T-cell restricted intracellular antigen 1
RelA	transcription factor p65 A subunit
RelB	transcription factor p65 B subunit
TGF-B	transforming growth factor beta
TNF-α	tumor necrosis factor alpha
OX-40L	tumor necrosis factor receptor superfamily member 4 ligand
tP53	tumor protein 53
VCP	valosin-containing protein
VEGF	vascular endothelial growth factor
WWOX	WW domain containing oxidoreductase
YB-1	y box binding protein 1
ZNF 687	zinc finger protein 687
